# Germline multigene panel testing revealed a *BRCA2* pathogenic variant in a patient with suspected Lynch syndrome

**DOI:** 10.1007/s13691-020-00449-9

**Published:** 2020-10-09

**Authors:** Tomoko Yoshihama, Akira Hirasawa, Kokichi Sugano, Teruhiko Yoshida, Mineko Ushiama, Arisa Ueki, Tomoko Akahane, Yoshiko Nanki, Kensuke Sakai, Takeshi Makabe, Wataru Yamagami, Nobuyuki Susumu, Kaori Kameyama, Kenjiro Kosaki, Daisuke Aoki

**Affiliations:** 1 Department of Obstetrics and Gynecology, Keio University School of Medicine, Tokyo, Japan; 2 Department of Clinical Genomic Medicine, Graduate School of Medicine, Dentistry and Pharmaceutical Sciences, Okayama University, 2-5-1 Shikata-cho, Kita-ku, Okayama, 700-8558 Japan; 3 Center for Medical Genetics, Keio University School of Medicine, Tokyo, Japan; 4 Oncogene Research Unit/Cancer Prevention Unit, Tochigi Cancer Center Research Institute, Tochigi, Japan; 5 Department of Genetic Medicine and Services, National Cancer Center Hospital, Tokyo, Japan; 6 Department of Obstetrics and Gynecology, International University of Health and Welfare, Chiba, Japan; 7 Department of Pathology, Showa University Northern Yokohama Hospital, Kanagawa, Japan

**Keywords:** *BRCA2*, Hereditary breast and ovarian cancer, Multigene panel testing, Genetic counseling, Lynch syndrome

## Abstract

There has been a rapid advance in germline multigene panel testing by next-generation sequencing, and it is being widely used in clinical settings. A 56-year-old woman suspected of having Lynch syndrome was identified as a *BRCA2* pathogenic variant carrier by multigene panel testing. The patient was diagnosed with endometrial cancer at the age of 39 years, and total laparoscopic hysterectomy and bilateral salpingectomy were performed at the age of 49 years; however, bilateral oophorectomy was not performed at that time. As she had a family history of colorectal cancer and a history of endometrial cancer, Lynch syndrome was suspected. However, germline multigene panel testing revealed a pathogenic *BRCA2* variant rather than pathogenic variants in mismatch repair genes. In this case, with conventional genetic risk assessment, we were unable to determine whether the patient had a high risk of hereditary breast and ovarian cancer; thus, germline multigene panel testing may provide valuable information to improve disease management strategies for patients in clinical settings. Particularly, germline multigene panel testing may be useful for detecting hereditary tumor syndromes if a patient does not present with a typical family history of cancer.

## Introduction

During recent years, there has been a rapid advance in genetic testing techniques such as next-generation sequencing, and they are being increasingly used in clinical practice, rather than conventional single-gene analysis [1]. Multigene panel testing by next-generation sequencing enables simultaneous analysis of multiple genes of interest at a lower cost than conventional techniques [2–5]. In some cases, this tool has provided valuable information that has enabled to change clinical management strategies for patients [6]. Here, we present a case of an individual suspected of having Lynch syndrome, but multigene panel testing revealed that the patient was a carrier of a *BRCA2* pathogenic variant.

## Case report

### Clinical history

**Fig. 1:**
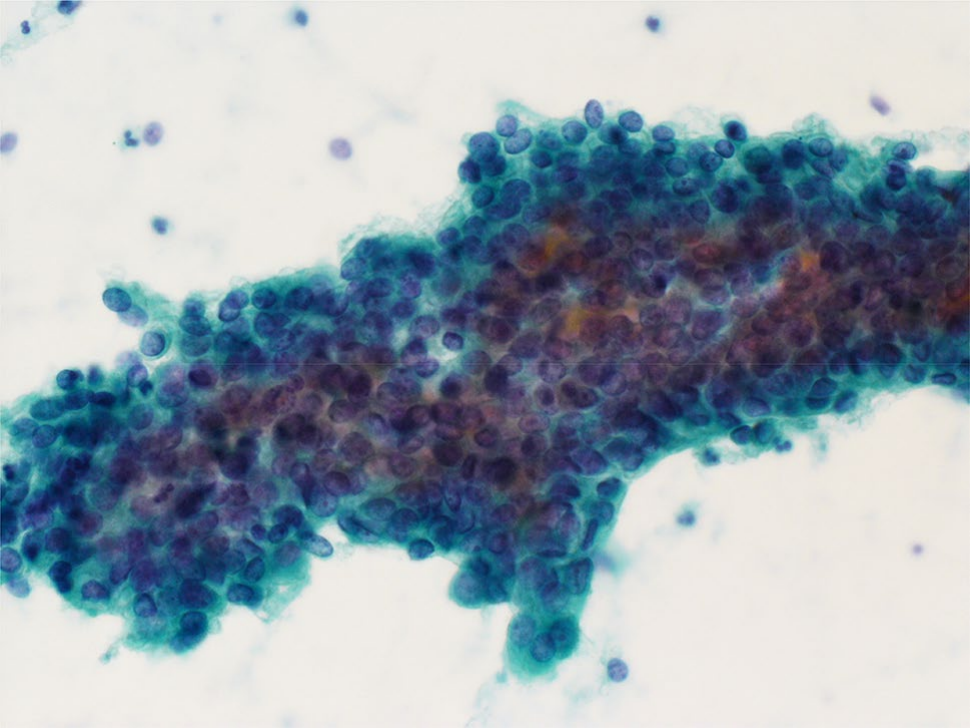
Cytology of the endometrium. Cell clusters exfoliated from the endometrium showed considerable nuclear overlapping. The nuclei were irregular and hyperchromatic and had coarse chromatin. Based on these findings, endometrioid adenocarcinoma was suspected

**Fig. 2:**
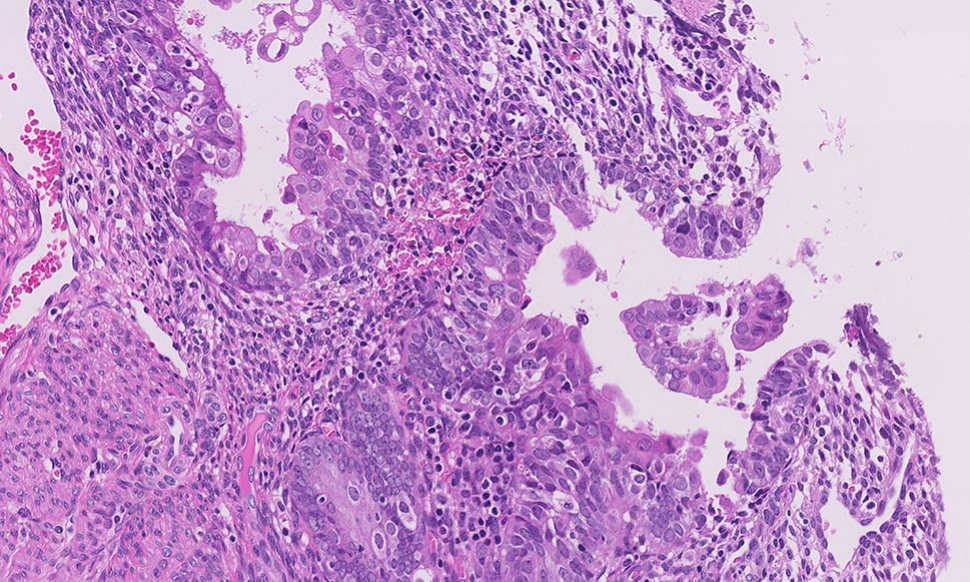
Microscopic analysis of the endometrial specimen stained with Hematoxylin and Eosin. The endometrial glands showed a small non-villous papillary architecture and were lined by cuboidal or columnar cells with pale eosinophilic cytoplasm. The nuclei were round and enlarged, without appreciable atypia

**Fig. 3:**
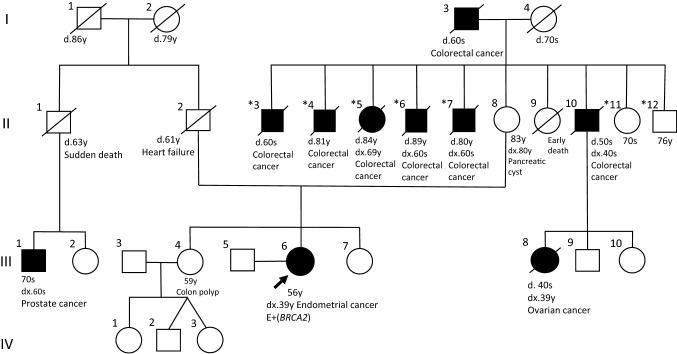
Family tree. The patient in this case is indicated with an arrow. The black squares and circles indicate family members with a history of malignancy. d., age at death; dx., age at diagnosis. II-3, -7, -11, and -12 have two children each, and II-4, -5, and -6 have three children each, none of them were affected by any related tumors (data not shown)

The patient, who had no particular history, was diagnosed with stage Ia endometrial cancer (FIGO 1988) when she was 39 years old. Her height was 158 cm and weight was 56 kg; the body mass index (BMI) was 22.4. The histological subtype was endometrioid carcinoma Grade 1. As the patient was never pregnant and hoped to preserve her fertility, medroxyprogesterone acetate therapy was carried out, instead of hysterectomy. As her tumor recurred when she was 49 years old, as revealed by the cytological analysis (Fig. 1), total laparoscopic hysterectomy and bilateral salpingectomy were performed, but the bilateral ovaries were not resected according to her wish. The final histopathological diagnosis was endometrioid carcinoma Grade 1 with no myometrial invasion (Fig. 2). Figure 3 shows the patient’s family tree. On the maternal side, six of her uncles and aunts (II-3, 4, 5, 6, 7, and 10), as well as her grandfather (I-3), had a history of colorectal cancer. In addition, one of her maternal cousins (III-8) had ovarian cancer and a paternal cousin (III-1) had prostate cancer.

### Genetic counseling

The attending physician suggested hereditary involvement, and genetic counseling was provided based on informed consent when she was 56 years old. First, she was suspected of having Lynch syndrome because of her history of endometrial cancer and family history of colorectal cancer. However, we suspected Lynch syndrome, as she fulfilled neither the Amsterdam criteria II nor revised Bethesda guidelines; moreover, she was not affected by colorectal cancer, and her first-degree relatives (specifically, her mother) had no history of related tumors. Thus, this patient did not show typical features of Lynch syndrome, although a genetic factor was assumed to be functional in the background. Therefore, germline panel testing using OncoGuide NCC Oncopanel System FC v. 1.0 (Table 1) (Agilent, Tokyo, Japan) was performed in addition to microsatellite instability testing and mismatch repair protein immunohistochemistry of endometrial specimens.

**Table 1:** List of 121 genes analyzed using the NCC oncopanel system FC v. 1.0

*AIP*	*BRCA1*	*CYLD*	*FANCA*	*GALNT12*	*MITF*	*PALLD*	*PTCH2*	*SDHA*	*TGFBR2*	*XRCC2*
*AKT1*	*BRCA2*	*DDB2*	*FANCB*	*GEN1*	*MLH1*	*PHOX2B*	*PTEN*	*SDHAF2*	*TMEM127*	
*ALK*	*BRIP1*	*DICER1*	*FANCC*	*GOLGA5*	*MLH3*	*PIK3CA*	*RAD50*	*SDHB*	*TP53*	
*APC*	*CDC73*	*EGFR*	*FANCD2*	*GREM1*	*MRE11A*	*PMS2*	*RAD51B*	*SDHC*	*TP53BP1*	
*ATM*	*CDH1*	*EGLN1*	*FANCE*	*HOXB13*	*MSH2*	*POLD1*	*RAD51C*	*SDHD*	*TSC1*	
*ATR*	*CDK4*	*ELAC2*	*FANCF*	*KIF1B*	*MSH3*	*POLE*	*RAD51D*	*SLX4*	*TSC2*	
*AXIN1*	*CDKN1B*	*EPCAM*	*FANCG*	*KIT*	*MSH6*	*POLH*	*RB1*	*SMAD4*	*TSHR*	
*AXIN2*	*CDKN2A*	*ERBB2*	*FANCI*	*KRAS*	*MUTYH*	*PPM1D*	*RBM15*	*SMARCB1*	*VHL*	
*BAP1*	*CHEK1*	*ESR1*	*FANCL*	*MAX*	*NBN*	*PRKAR1A*	*RECQL4*	*SMARCE1*	*WRN*	
*BARD1*	*CHEK2*	*EXT1*	*FANCM*	*MC1R*	*NF1*	*PRKDC*	*RET*	*STK11*	*WT1*	
*BLM*	*CTNNA1*	*EXT2*	*FH*	*MEN1*	*NF2*	*PRSS1*	*RHBDF2*	*SUFU*	*XPA*	
*BMPR1A*	*CTNNB1*	*FAM175A*	*FLCN*	*MET*	*PALB2*	*PTCH1*	*RNF139*	*TERT*	*XPC*	

## Results

**Fig. 4:**
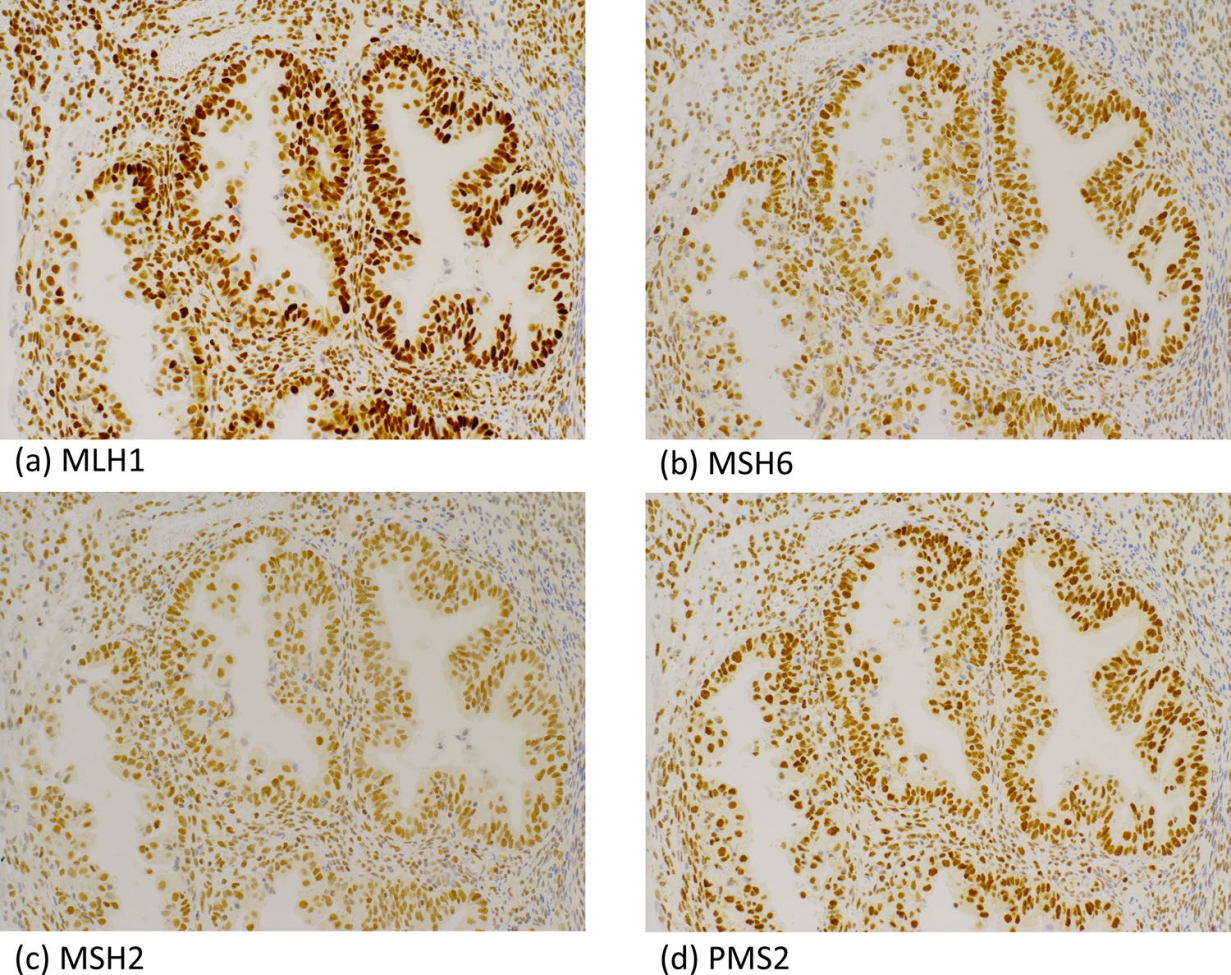
Immunohistochemical staining of mismatch repair proteins. The tumor cell nuclear expression of MLH1, MSH6, MSH2, and PMS2 was similar to that in the surrounding stromal cells

The stability of microsatellites and the expression of mismatch repair proteins were not decreased, according to microsatellite instability testing and immunohistochemistry analysis, respectively (Fig. 4). Germline multigene panel testing revealed a *BRCA2* pathogenic variant (exon13: c.C6952T, p.R2318X).

### Clinical management

The patient had a *BRCA2* pathogenic variant, and information regarding the future risk of breast, ovarian, and pancreatic cancers was collected. The option of prophylactic resection of either the mammaries (by risk-reducing mastectomy) or ovaries was recommended according to management guidelines, such as those from the National Comprehensive Cancer Network. Genetic counseling for other family members considered to be at risk was also considered. This patient is currently 56 years old, and to date, she has not undergone prophylactic surgery. Instead, she is under surveillance for breast, ovarian, and pancreatic cancers in our hospital.

## Discussion

In this study, a *BRCA2* pathogenic variant was detected in the patient. The *BRCA2* exon 13 variant c.C6952T, p.R2318X was identified as a pathogenic variant according to previous studies [7–9]. The frequency of this variant has been reported to be 0.44% in Japanese patients with breast cancer [8], and this variant has also been reported in Japanese patients with ovarian cancer [9]. Here, when reviewing the family tree (Fig. 3) with this genetic information, it was apparent that one maternal cousin (III-8) had a history of ovarian cancer, one paternal cousin (III-1) had prostate cancer, and no family member on either side had breast cancer. As prostate cancer is also related to hereditary breast and ovarian cancer (HBOC), it is difficult to determine the family member from whom the *BRCA2* variant was derived. Additional examination in other family members should be considered to clarify hereditary tumor involvement and perform segregation analysis.

One of the most important clinical considerations in this case was that the bilateral ovaries of the patient were not resected. In addition, the fallopian tubes were not pathologically examined in accordance with the SEE-FIM protocol at that time. When total laparoscopic hysterectomy and bilateral salpingectomy were performed to treat her endometrial cancer, there was no information regarding her *BRCA2* pathogenic variant. On the contrary, this patient did not meet the *BRCA1/2* testing criteria of the National Comprehensive Cancer Network Guidelines (v. 3, 2019), as she had no history of breast or ovarian cancer and only one of her cousins had ovarian cancer. Therefore, it was difficult to evaluate the risk of HBOC in this patient using only the personal and family histories. In contrast, if multigene panel testing had been performed before surgery, this patient could have opted bilateral salpingo-oophorectomy based on the future ovarian cancer risk; moreover, the fallopian tubes should have been appropriately examined.

Thus, multigene panel testing may change disease management in clinical settings based on results that cannot be obtained from either conventional risk assessments or single-gene analysis. Indeed, previous studies have indicated that multigene panel testing can increase the detection rate of any pathogenic variant (including non-*BRCA*1/2 variants, such as Lynch syndrome genes) in patients suspected with HBOC, and this can alter clinical management strategies for cancers [6, 10]. Kurian et al. also reported that *BRCA1/2*-only testing is being replaced by multiple-gene sequencing for patients with breast cancer [11]. Furthermore, some previous studies identified *BRCA2* pathogenic variants in patients with colorectal cancer based on the age at diagnosis or family history of colorectal cancers [12, 13]. Here, we suggest that multigene panel testing is also useful for improving the clinical management strategies of patients who had been primarily suspected to have Lynch syndrome and not HBOC.

However, several issues should be considered when performing multigene panel testing. For example, there may be some differences in the targeted genes or variant annotations among the commercially available tests. The possibility of identifying variants of unknown significance or variants in genes that are not thought to be clinically actionable should also be considered. Therefore, single gene analysis could be considered for some hereditary tumors, which have characteristic phenotypes, such as, Peutz–Jeghers syndrome. Individuals providing genetic counseling should be familiar with updated information regarding the problems mentioned above.

Regarding the relationship between the *BRCA1/2* variant and endometrial cancer, Shu et al. reported that the risk of serous/serous-like endometrial carcinoma increased in *BRCA1* variant-positive women [14]. In our case, the patient had a *BRCA2* pathogenic variant, and the endometrial cancer histology was endometrioid carcinoma. Therefore, it is unclear whether the *BRCA2* variant affected the etiology of endometrial cancer. On the contrary, this patient did not have typical clinical features of endometrial cancer such as obesity or a history of diabetes mellitus. Although nulliparity could be a risk factor, it is obscure how the patient developed endometrial cancer in her thirties.

In conclusion, we present a case in which multigene panel testing revealed a *BRCA2* pathogenic variant in a patient who had been suspected to have Lynch syndrome, rather than HBOC. Clinicians should take detailed history of patients and their families, particularly when planning a surgery and should carefully choose an appropriate genetic testing tool that may confirm or alter the clinical management strategy.
